# GEOfetch: a command-line tool for downloading data and standardized metadata from GEO and SRA

**DOI:** 10.1093/bioinformatics/btad069

**Published:** 2023-03-01

**Authors:** Oleksandr Khoroshevskyi, Nathan LeRoy, Vincent P Reuter, Nathan C Sheffield

**Affiliations:** Center for Public Health Genomics, School of Medicine, University of Virginia, Charlottesville, VA 22908, USA; Center for Public Health Genomics, School of Medicine, University of Virginia, Charlottesville, VA 22908, USA; Department of Biomedical Engineering, School of Medicine, University of Virginia, Charlottesville, VA 22904, USA; Center for Public Health Genomics, School of Medicine, University of Virginia, Charlottesville, VA 22908, USA; Center for Public Health Genomics, School of Medicine, University of Virginia, Charlottesville, VA 22908, USA; School of Data Science, University of Virginia, Charlottesville, VA 22904, USA; Department of Biomedical Engineering, School of Medicine, University of Virginia, Charlottesville, VA 22904, USA; Department of Public Health Sciences, School of Medicine, University of Virginia, Charlottesville, VA 22908, USA; Department of Biochemistry and Molecular Genetics, School of Medicine, University of Virginia, Charlottesville, VA 22908, USA

## Abstract

**Motivation:**

The Gene Expression Omnibus has become an important source of biological data for secondary analysis. However, there is no simple, programmatic way to download data and metadata from Gene Expression Omnibus (GEO) in a standardized annotation format.

**Results:**

To address this, we present GEOfetch—a command-line tool that downloads and organizes data and metadata from GEO and SRA. GEOfetch formats the downloaded metadata as a Portable Encapsulated Project, providing universal format for the reanalysis of public data.

**Availability and implementation:**

GEOfetch is available on Bioconda and the Python Package Index (PyPI).

## 1 Introduction

The increase in biological data has led to challenges in data storage, sharing and reuse. The largest repositories of sequencing data include the Gene Expression Omnibus (GEO) (Barrett *et al.*, 2013) and Sequence Read Archive (SRA) (Katz *et al.*, 2022), which hold data and metadata from hundreds of thousands of biological samples. These databases are a major data resource; however, it can be time-consuming to download and restructure results programatically, creating a barrier for data reuse.

Some recent tools have addressed this issue by parsing sample metadata to create a more digestible structure (Davis and Meltzer, 2007; Gumienny, 2019). Another approach has been to reprocess all the GEO metadata and make it searchable in a web interface (Chen *et al.*, 2019). Yet other tools are focused on fetching metadata and URLs to files either from SRA specifically (Choudhary, 2019; Ewels *et al.*, 2020) or across databases (Gálvez-Merchán *et al.*, 2022). These tools help make the data and metadata more reusable, but they are limited in several ways; we needed a more flexible system with a command-line interface, ability to find and retrieve many projects simultaneously, pre-download filter options, duplication detection, handling both metadata and data, and standardized output usable across languages.

Here, we present GEOfetch, a Python package and command-line interface for retrieving data and metadata from GEO. Users provide a list of GEO or SRA accessions and GEOfetch will download, parse, and restructure the metadata for universal downstream analysis. GEOfetch downloads either processed data from GEO or raw data from SRA and then creates a standardized sample metadata table following the Portable Encapsulated Project (PEP) specification (Sheffield *et al.*, 2021). PEPs are a standardized, language-agnostic representation of sample metadata that facilitate downstream analysis, including deploying pipelines across samples, loading processed data into R or Python, or meta-analysis that spans projects (Sheffield *et al.*, 2021). GEOfetch provides options such as (i) downloading just metadata or metadata with data; (ii) if data are requested, whether to download raw or processed data; (iii) changing output location; (iv) whether to combine metadata into one table or split by accession; (v) whether to include GEO series (project-level) data, sample-level data or both; and (vi) other features described below. With these features, GEOfetch provides a powerful and standardized interface to data and metadata on GEO and SRA.

## 2 Results

**Fig. 1.**
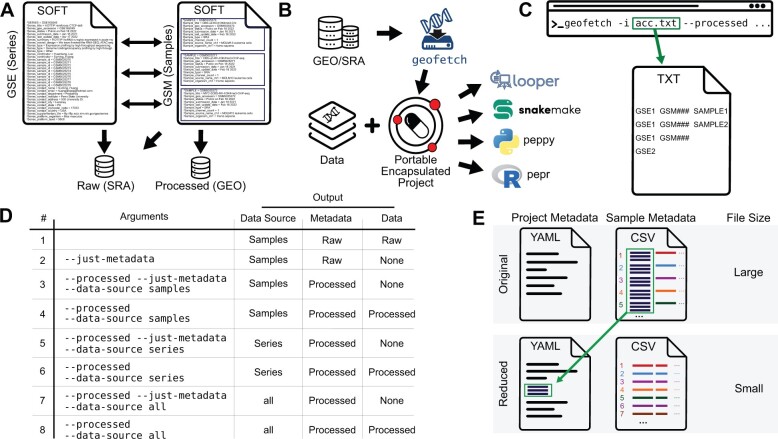
Unnumbered figure: GEOfetch overview. (**A**) GEO metadata has complex links to raw and processed data. (**B**) GEOfetch downloads data and metadata from GEO and SRA and produces a Portable Encapsulated Project. (**C**) GEOfetch command line accepts GEO accession IDs. (**D**) Table of arguments and corresponding GEOfetch behavior. (**E**) GEOfetch consolidates large, constant sample attributes into project metadata to reduce space use

The structure of metadata on GEO and SRA is complex (Fig. 1A). GEO data are attached to *samples*, which are organized into groups called *series*. Both samples and series contain metadata and may also link to processed data stored in GEO. In addition, samples may also link to raw data stored on SRA.

The metadata of both samples and series entities is stored in Simple Omnibus Format (SOFT). Samples may link to multiple series, and a series may contain multiple samples (Barrett *et al.*, 2013). GEOfetch handles downloading both data and metadata. For metadata, GEOfetch reads, filters, processes, and restructures the SOFT files to produce a PEP, making it easy to import metadata in Python or R using the peppy or pepr packages and facilitating analysis using PEP-compatible tools (Fig. 1B) (Mölder *et al.*, 2021; Sheffield *et al.*, 2021).

The workhorse of GEOfetch is its command-line interface. As input, a user simply provides one or more GEO accession ids (GSE###) as a command-line argument, and GEO will download all included samples. A user may also subset a GEO experiment by providing a file with three columns that correspond to GSE, GSM, and sample name, providing granular control over download (Fig. 1C).

GEOfetch can also be tuned to download different combinations of metadata, processed data from GEO, and raw data from SRA (Fig. 1D). This behavior is controlled with three arguments: By default, GEOfetch will download raw data from SRA, but the --*processed* flag downloads processed (GEO) data. By default, GEOfetch will download processed data from samples, not series; this may be modulated with the --*data-source* argument, with options *samples*, *series,* or *all*. Finally, the --*just-metadata* flag tells GEOfetch to download only metadata.

Because many GEO accessions have a variety of attached data, and users may be seeking only a particular data type, GEOfetch also provides filters to restrict the filenames of data actually downloaded. The--filter argument allows the user to provide a filename pattern as a regular expression, and GEOfetch will download only files with matching names. Users may also specify a maximum file size with the--filter-size argument to avoid downloading large files.

One challenge with metadata from GEO is long text fields that are constant across all samples, such as details of the sample protocol. To streamline output, GEOfetch can store large, constant sample attributes at the project level, instead of with individual samples (Fig. 1E). This approach reduces size of the metadata and improves readability. Users may also elect to discard such columns, rather than storing them in the project configuration file. These features are controlled through command-line arguments: --const-limit-project, const-limit-discard and --attr-limit-truncate.

GEOfetch also provides a Python API, allowing it to be integrated into Python software. GEOfetch can create Project and Sample metadata objects directly in memory. GEOfetch also provides a Finder class that retrieves a list of GSEs given a user search query. The Finder can search by date to identify recent changes in GEO, or use other advanced search terms. The GSE accessions retrieved by the Finder can then be provided to GEOfetch to download data and metadata.
